# Epstein–Barr virus-positive lymphoproliferative disorder manifesting as pulmonary disease in a patient with acute myeloid leukemia: a case report

**DOI:** 10.1186/s13256-021-02744-2

**Published:** 2021-03-28

**Authors:** Ritika Dutta, Susanna Y. Miao, Paul Phan, Sebastian Fernandez-Pol, Parveen Shiraz, Dora Ho, Gabriel N. Mannis, Tian Y. Zhang

**Affiliations:** 1 Department of Medicine, Division of Hematology, Cancer Institute, and Institute for Stem Cell Biology and Regenerative Medicine, Stanford University, Stanford, CA USA; 2 Stanford University School of Medicine, Stanford, CA USA; 3 Department of Medicine, Stanford University, Stanford, CA USA; 4 Department of Pathology, Stanford University, Stanford, CA USA; 5 Department of Medicine, Division of Blood and Marrow Transplantation, Stanford University, Stanford, CA USA; 6 Department of Medicine, Division of Infectious Diseases & Geographic Medicine, Stanford University, Stanford, CA USA

**Keywords:** Acute myeloid leukemia, Epstein–Barr virus, Post-transplant lymphoproliferative disorder, Hematopoietic stem cell transplant, Case report

## Abstract

**Background:**

Patients with lymphoproliferative disorders following hematopoietic stem cell transplant (HSCT) most commonly present with fever and lymphadenopathy within the first 5 months of transplant. Pulmonary post-transplant lymphoproliferative disorder (PTLD) is a particularly aggressive and rapidly progressive disease, with high morbidity and mortality. There are a very limited number of reported pulmonary PTLD cases following HSCT in patients with acute myeloid leukemia (AML). Early diagnosis and detection of pulmonary PTLD is critical given its high lethality. However, variable clinical presentations and nonspecific radiographic findings make pulmonary PTLD difficult to distinguish from other more common causes of pulmonary disease in AML patients.

**Case presentation:**

Here, we describe a 68-year-old Caucasian man who presented for salvage induction therapy following relapse of his AML after a haploidentical allogeneic HSCT 10 months earlier. He developed recurrent fevers, dry cough, and hypoxemia, with chest computed tomography (CT) showing bibasilar consolidations and increased nodularity without increased lymphadenopathy. His symptoms initially improved with antibiotic and antifungal therapy, but his follow-up chest CT showed progression of disease despite symptomatic improvement. Epstein–Barr virus (EBV) was detected in his blood by polymerase chain reaction (PCR), and a lung biopsy revealed monomorphic PTLD with B cells positive for EBV. Unfortunately, the patient’s condition rapidly deteriorated, and he passed away prior to treatment initiation.

**Conclusions:**

To our knowledge, this is the first reported case of an AML patient developing pulmonary PTLD relatively late in his post-transplant course in the setting of relapsed disease and salvage therapy. Pulmonary PTLD, a rare but highly lethal disorder, can imitate the symptoms and radiographic findings of pneumonia, a common diagnosis in immunocompromised AML patients. This case illustrates the importance of considering pulmonary PTLD in the differential diagnosis for pulmonary disease in AML patients with a history of HSCT, especially in the setting of progressive radiographic findings despite broad antibacterial and antifungal therapy. Further, our case demonstrates the importance of biopsy and uninterrupted EBV DNA monitoring in the definitive diagnosis of PTLD, given nonspecific symptomatology and radiographic findings.

## Background

Epstein–Barr virus-associated post-transplant lymphoproliferative disorders (EBV-PTLDs) are a heterogenous group of rare, but potentially fatal, diseases that can develop following allogeneic hematopoietic stem cell transplant (allo-HSCT) or solid-organ transplant (SOT) [1, 2]. *De novo* EBV infection or EBV reactivation in the setting of compromised T-cell immunity can lead to transformation and immortalization of lymphocytes, most commonly B cells, subsequently resulting in uncontrolled proliferation. Compared with SOT, post-HSCT PTLD has a more aggressive clinical course, inferior response to therapy, and shortened overall survival [3]. The overall incidence of PTLD in allo-HSCT recipients ranges from 0.5 to 6% and is particularly high in the pediatric population [1, 4–9]. Most of these cases are EBV-related, donor-derived, and develop within 1 year of HSCT, with the highest incidence occurring in the first 5 months following transplant [1, 5]. Known risk factors for PTLD include several transplant-associated characteristics, including reduced-intensity conditioning, T-cell depletion of the graft, inclusion of anti-thymocyte globulin (ATG) for graft-versus-host disease (GVHD) prophylaxis, mismatch of human leukocyte antigens, and severe GVHD [5, 6].

Clinically, patients with EBV-PTLD most commonly present with fever, lymphadenopathy, and/or infectious mononucleosis symptoms. Patients can also develop extranodal disease, most commonly in the liver, gastrointestinal tract, spleen, central nervous system, and lungs [1, 3, 9]. Early detection of EBV in the blood by polymerase chain reaction (PCR) has greatly improved outcomes for EBV-PTLD, allowing for timely treatment with rituximab. Guidelines from the European Conference on Infections in Leukaemia recommend weekly screening for EBV-DNA in the blood (EBV DNAemia) for at least 4 months following transplant [10]. The time from initial detection of EBV DNAemia to clinical manifestations of EBV-associated disease is typically very short, with a median of 7 days, highlighting the rapidly progressive nature of the disease [11]. Despite appropriate treatment, mortality for EBV-PTLD remains approximately 30% [10].

Pulmonary PTLD following allo-HSCT is a poorly understood and particularly aggressive form of the disease, with a median reported duration between diagnosis and death of 5 days [7]. Thus, early clinical suspicion and detection is crucial to improve survival. However, this remains difficult because pulmonary PTLD is scarcely described in medical literature and has a wide range of presentations, from localized disease to disseminated lymphoma with multiorgan dysfunction [7]. From our review of the literature, there have been few reports of pulmonary PTLD cases arising in patients with adult acute myeloid leukemia (AML) following allo-HSCT [7, 12]. Most reported cases occurred in younger patients (<40) with known risk factors, such as severe GVHD and ATG immunosuppression, and presented within 2 months of transplant. Here, we report a unique case of a 68-year-old man who developed pulmonary PTLD 10 months following his allo-HSCT while receiving salvage therapy for relapsed AML.

## Case presentation

**Fig. 1:**
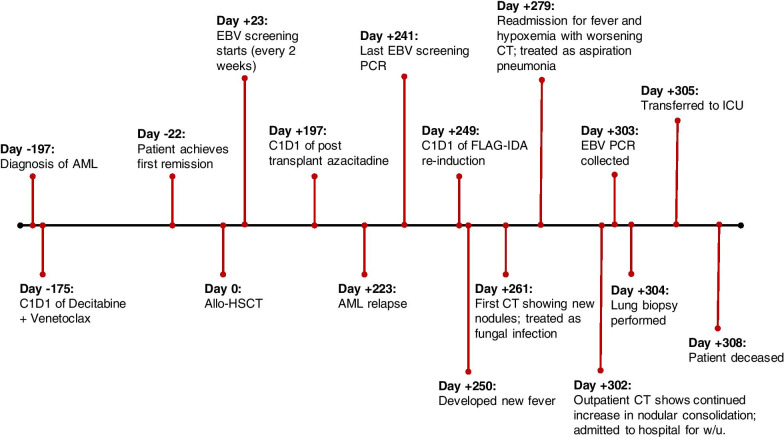
Timeline of disease. Timeline depicting the major events during the patient’s disease course as described in the text. *AML* acute myeloid leukemia, *CxDy* Cycle x, Day y of chemotherapy treatment, *EBV* Epstein–Barr virus, *Allo-HSCT* allogeneic hematopoietic stem cell transplant, *PCR* polymerase chain reaction, *FLAG-IDA* fludarabine, cytarabine, idarubicin, and G-CSF, *CT* computed tomography, *ICU* intensive care unit, *W/u* workup

A 68-year-old Caucasian man with a past medical history of Crohn’s disease, hypertension, type 2 diabetes mellitus, obstructive sleep apnea, and atrial fibrillation, as well as noncontributory psychosocial or family history, was diagnosed with adverse risk, therapy-related AML after presenting with progressive cytopenias (Fig. 1). His bone marrow (BM) biopsy was remarkable for 27% blasts in a background of trilineage dysplasia with a karyotype of del(5q), del(20q), and trisomy 8. Targeted next-generation sequencing revealed a pathogenic somatic mutation in *TP53* as well as variants of unknown significance in *ASXL1*, *FAS*, and *PIK3CA*. AML with myelodysplasia-related changes was diagnosed based on his *TP53* mutation and complex karyotype. He achieved first complete remission (CR1) with no measurable residual disease (minimal residual disease [MRD]-negative) by multiparametric flow cytometry after 5 cycles of decitabine/venetoclax combination therapy. His initial chemotherapy course was complicated by the development of pulmonary nodules within 1 month of treatment initiation, concerning for invasive fungal infection (without microbiologic confirmation). He was treated with posaconazole with subsequent resolution. He then underwent haploidentical HSCT with peripheral blood stem cells from a relative using a reduced-intensity conditioning regimen consisting of fludarabine, cyclophosphamide, and total-body irradiation (Flu/CY/TBI). GVHD prophylaxis consisted of post-transplant cyclophosphamide, tacrolimus, and mycophenolate mofetil. His post-transplant course was complicated by *Morganella morganii* bacteremia, a *Demodex* skin infection, and grade II-III acute GVHD of the skin. Peripheral blood chimerism on day +90 post-transplant demonstrated successful engraftment, with 99–100% donor cells, and a BM biopsy showed continued MRD-negative CR.

Unfortunately, a repeat BM biopsy 6 months following transplant showed a 0.2% abnormal blast population by flow cytometry. To augment the graft-versus-leukemia effect, the patient received two cycles of post-allo-HSCT azacitidine, and immunosuppressants were rapidly tapered. However, a BM biopsy on day +223 following transplant revealed relapsed AML with a 10% blast population exhibiting complex cytogenetics and a pathogenic mutation in *TP5*3, as well as variants of unknown significance in *FAS*, *PIK3CA*, *SETD2*, and *TET2*, with mixed chimerism. The patient was then admitted to the hospital for salvage induction with FLAG-IDA (fludarabine, cytarabine, idarubicin, and granulocyte colony-stimulating factor [G-CSF]).

**Fig. 2:**
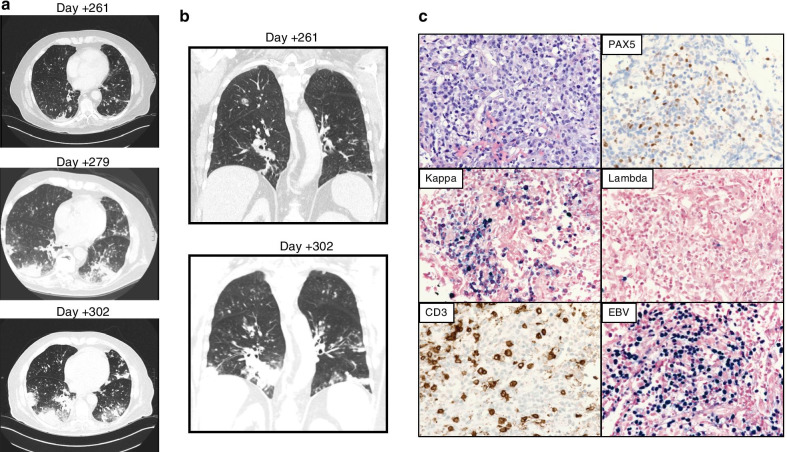
Images of patient’s Epstein–Barr virus-positive lymphoproliferative disease (EBV-PTLD). **a** Computed tomography (CT) images of the lung bases (axial view) showing progression of disease from day +261 (top image) to day +279 (middle image) to day +302 (bottom image). CT images show bibasilar progressive confluence of consolidations and new nodules. **b** CT images of lungs (coronal view) showing increased nodularity and consolidation from day +261 (top image) to day +302 (bottom image). **c** Histology images from the fine needle aspiration/core biopsy of right lower lobe performed on day +304 showing a uniform population of medium to large Pax5-partially positive B cells that are kappa light chain-restricted and EBV-positive by *in situ* hybridization for EBV-encoded ribonucleic acid (RNA)

During the hospital admission for FLAG-IDA induction (day +249 post-transplant), the patient developed febrile neutropenia with throat irritation and an accompanying cough. His physical exam was notable for decreased breath sounds and bilateral wheezes. He was found to have respiratory syncytial virus and started on ribavirin (oral) with meropenem and levofloxacin due to persistent fevers and a new oxygen requirement. A chest computed tomography (CT) scan (day +261) scan demonstrated new nodular opacities with surrounding ground glass along with bibasilar consolidations (Fig. 2a, b). Due to concern for possible pulmonary fungal infection, bronchoscopy was performed. Bronchoalveolar lavage (BAL) was only remarkable for an *Aspergillus* galactomannan index >2; serum cell-free fungal DNA was negative. Given radiographic findings suspicious for a fungal infection and a positive BAL fungal marker, antifungal treatment was started using intravenous liposomal amphotericin B (AmBisome^®^). His fever curve and oxygen requirement improved after initiation of antifungal therapy and count recovery. He was then discharged on posaconazole, as well as prophylactic acyclovir and trimethoprim/sulfamethoxazole. However, he was readmitted shortly afterwards with low-grade fever, rigors, chills, cough, and hypoxemia. A chest/abdomen/pelvis CT scan (day +279) was remarkable for increased confluence of consolidations in both lower lobes and additional nodules throughout both lungs (Fig. 2a). The patient was treated for a presumed aspiration pneumonia with broad-spectrum antibiotics, which led to resolution of the fevers and improvement of the cough.

Two weeks following discharge, outpatient CT chest (day +302) imaging revealed progressive bronchocentric and basilar-predominant nodular consolidations despite continued treatment with posaconazole (Fig. 2a, b). Additionally, the patient continued to experience a persistent cough with clear sputum and without associated dyspnea or B symptoms. He was readmitted to the hospital for expedited workup of the CT findings, with concern for a persistent invasive fungal infection. EBV-PCR was ordered (day +303), and fine needle aspiration and core biopsy of the right lower lobe were performed (day +304). Unfortunately, the patient developed increased work of breathing and altered mental status, necessitating transfer to the intensive care unit (ICU) (day +305).

His EBV deoxyribonucleic acid polymerase chain reaction (DNA PCR) results returned positive on day +308 at 23,142 IU/mL. Of note, the patient was screened every 2 weeks for EBV-DNA from day +23 post-transplant to day +241 with negative results. The lung biopsy revealed a uniform population of medium to large lymphoid cells with irregular nuclear contours, coarse chromatin, and scant cytoplasm. The final diagnosis was monomorphic PTLD with EBV-positive diffuse large B-cell lymphoma (Fig. 2c). By flow cytometry, the abnormal B-cell population expressed CD38 (bright), CD45, surface CD19, surface CD22 (77%), and CD20 (62%), and was negative for CD5, CD10, and surface light chain expression. *In situ* hybridization showed that the PTLD was EBV-positive and kappa light chain-restricted (Fig. 2c). A periodic acid-Schiff-diastase stain did not demonstrate any fungal organisms. Although preliminary results from the EBV DNA and lung biopsy became available on day +308, the patient’s condition had acutely deteriorated, requiring intubation and pressor support. Based on his family’s wishes, he was transitioned to comfort care and passed away on day +308.

## Discussion and conclusions

Pulmonary disease in a relapsed AML patient with a history of allo-HSCT has a broad differential, including infectious or noninfectious causes of pneumonia/pneumonitis, GVHD, and PTLD [13]. Symptomatology and imaging findings can be nonspecific and can overlap among these diagnoses. Typically, pulmonary PTLD is donor-derived, and patients present with fever, dyspnea and hypoxemia, diffuse lymphadenopathy, and systemic symptoms within 1–5 months following allo-HSCT [7, 13]. Radiographic features can include diffuse infiltrates in the basal and subpleural areas, pulmonary nodules, cavitation, peribronchial thickening, and reticulonodular shadowing [7, 13–15]. We describe a case of a 68-year-old man who developed pulmonary PTLD relatively late in his post-transplant course after tapering of immunosuppressive agents in the setting of AML salvage therapy with FLAG-IDA. He presented with fevers and a dry cough and later developed systemic symptoms. Although his symptoms were recurrent, they initially improved with antibiotic and antifungal therapy during each hospitalization, suggesting the possibility of a concomitant infectious process. He also did not demonstrate any new lymphadenopathy by physical exam or CT imaging during progression of his PTLD. Combined with his neutropenia and CT findings of bibasilar consolidations and increased nodularity, his clinical presentation was initially concerning for a pneumonia. Clinical suspicion for PTLD should be heightened in patients who have progressive radiographic pulmonary disease despite appropriate antifungal and antibacterial treatment. EBV-PCR can then be obtained to assess for EBV DNAemia as a marker for PTLD. Of note, our patient developed EBV DNAemia in the 62-day window between his last negative screen on day +241 post-transplant and the positive sample tested on day +303. It is possible that the new pulmonary nodules seen on his CT scan on day +261 represented the initial PTLD lesions, but this cannot be confirmed with certainty. Interestingly, our patient developed PTLD in the setting of FLAG-IDA salvage therapy after weaning of his transplant immunosuppressive regimen. This raises the possibility that chemotherapy may have contributed to his immunosuppressed state and development of lymphoproliferative disease [16]. Consistent with prior reports, this case reinforces the recommendations for frequent screening given the rapid clinical course of EBV-PTLD after the initial detection of EBV DNAemia [1, 7, 9]. Furthermore, given the variable findings of PTLD on radiographic imaging, this case highlights the importance of obtaining a lung biopsy for definitive diagnosis of PTLD to ensure that appropriate therapy can be initiated in a timely manner [2]. Although rituximab has been shown to be an efficacious therapy for EBV-PTLD, mortality in pulmonary PTLD patients remains high despite treatment, underlining a need for further research into the pathogenesis and therapeutic approaches for pulmonary PTLD. Newly available therapies that target other antigens such as CD19 (blinatumomab) and CD22 (inotuzumab) warrant study to see whether outcomes can be improved.

In conclusion, this case provides important anecdotal evidence to support the inclusion of pulmonary PTLD in the differential diagnosis of pulmonary disease in AML patients with a prior history of allo-HSCT. To our knowledge, this is the first reported case of an AML patient developing pulmonary PTLD relatively late in their post-transplant course in the setting of relapsed disease and salvage therapy. Our case highlights the importance of biopsy and frequent EBV DNA monitoring in the definitive diagnosis of PTLD, given nonspecific symptomatology and radiographic findings that can imitate other causes of pulmonary disease in AML patients.

## Data Availability

Data sharing is not applicable as no data sets were generated or analyzed during the study.

## Abbreviations

Allo-HSCT: Allogeneic hematopoietic stem cell transplant
AML: Acute myeloid leukemia
ATG: Anti-thymocyte globulin
BAL: Bronchoalveolar lavage
BM: Bone marrow
CR: Complete remission
CT: Computed tomography
EBV: Epstein–Barr virus
EBV-PTLD: Epstein–Barr virus-associated post-transplant lymphoproliferative disorder
FLAG-IDA: Fludarabine, cytarabine, idarubicin, and granulocyte colony-stimulating factor (G-CSF)
GVHD: Graft-versus-host disease
MRD: Minimal residual disease
PCR: Polymerase chain reaction
PTLD: Post-transplant lymphoproliferative disease/disorder
SOT: Solid organ transplant
